# Supplementary material for: Dual Effect of Tryptamine on Prostate Cancer Cell Growth Regulation: A Pilot Study
Source: Int J Mol Sci. 2022 Sep 21;23(19):11087. doi: 10.3390/ijms231911087 (PMC9569450; doi:10.3390/ijms231911087)
Supplement: Supplementary file 1 [file ijms-23-11087-s001.zip › ijms-1910517-supplementary.pdf]

Supporting Information

Table of Contents

Figure S1. Cell viability of PC-3 cell treated with various concentrations of tryptamine for 24 h.

Figure S2. Liver function test results and effects of tryptamine and 3-HAA treatments on the tumor growth of PC-3 xenograft mouse model. (A and B) Appearance and aspartate aminotransferase (AST, U/L) value of the liver of PC-3 xenograft mice received intraperitoneal administration of tryptamine (80 mg/kg, n = 6) and intratumoral administration of tryptamine (1 mg/100 mm^3, n = 5), respectively. Size of the tumors of PC-3 xenograft mice received intratumoral administration of (C and E) tryptamine (1 mg/100 mm^3) and 3-HAA (200 mg/kg) and intraperitoneal administration of (D and F) tryptamine (80 mg/kg) and 3-HAA (200 mg/kg), respectively.

Figure S3. Heatmap overview of altered PC-3 cell metabolic profiles in LC-MS (A) positive and (B) negative ion modes before and after tryptamine treatment.

Figure S4. MS/MS analyses of the discriminant metabolites in panels.

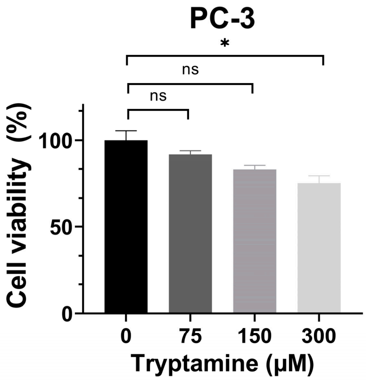

Figure S1. Cell viability of PC-3 cell treated with various concentrations of tryptamine for 24h.
* $p < 0.05$, ** $p < 0.01$, *** $p < 0.001$. n = 3.

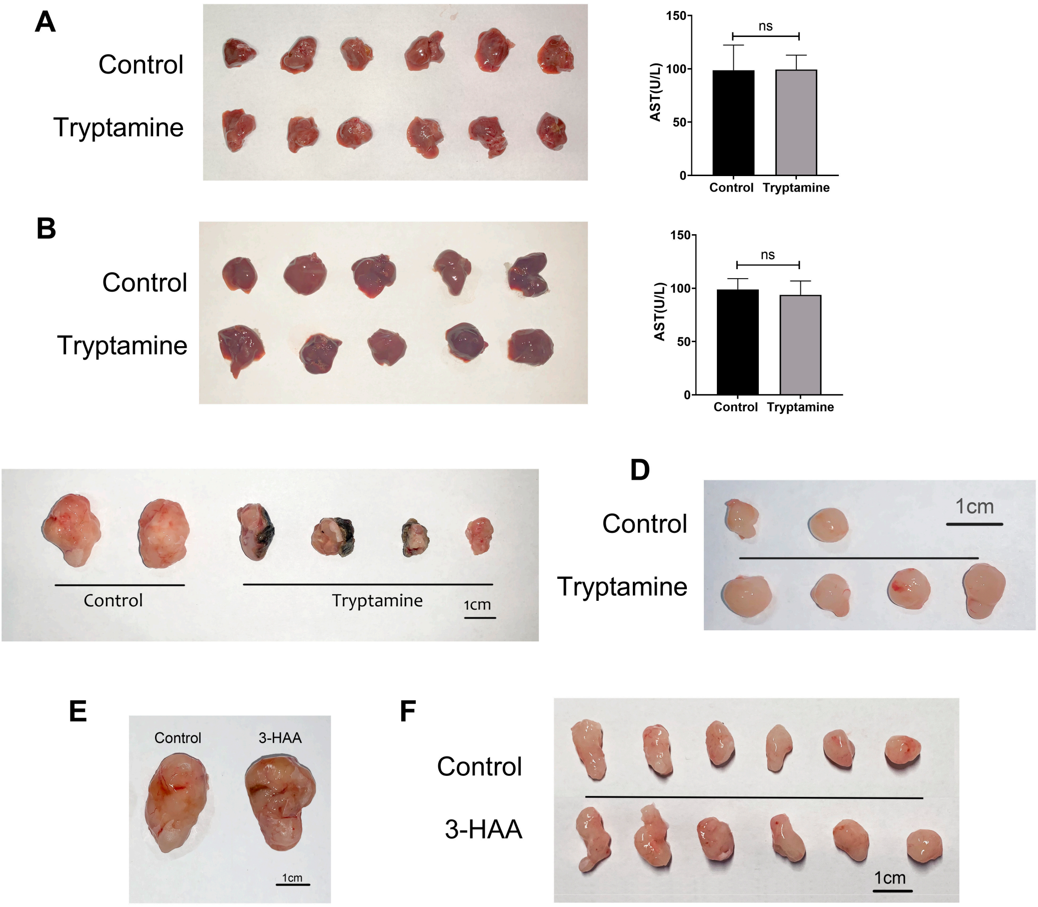

Figure S2. Liver function test results and effects of tryptamine and 3-HAA treatments on the tumor growth of PC-3 xenograft mouse model. (A and B) Appearance and aspartate aminotransferase (AST, U/L) value of the liver of PC-3 xenograft mice received intraperitoneal administration of tryptamine (80 mg/kg, n = 6) and intratumoral administration of tryptamine (1 mg/100 mm^3, n = 5), respectively. Size of the tumors of PC-3 xenograft mice received intratumoral administration of (C and E) tryptamine (1 mg/100 mm^3) and 3-HAA (200 mg/kg) and intraperitoneal administration of (D and F) tryptamine (80 mg/kg) and 3-HAA (200 mg/kg), respectively. Data were represented as mean ± SD.

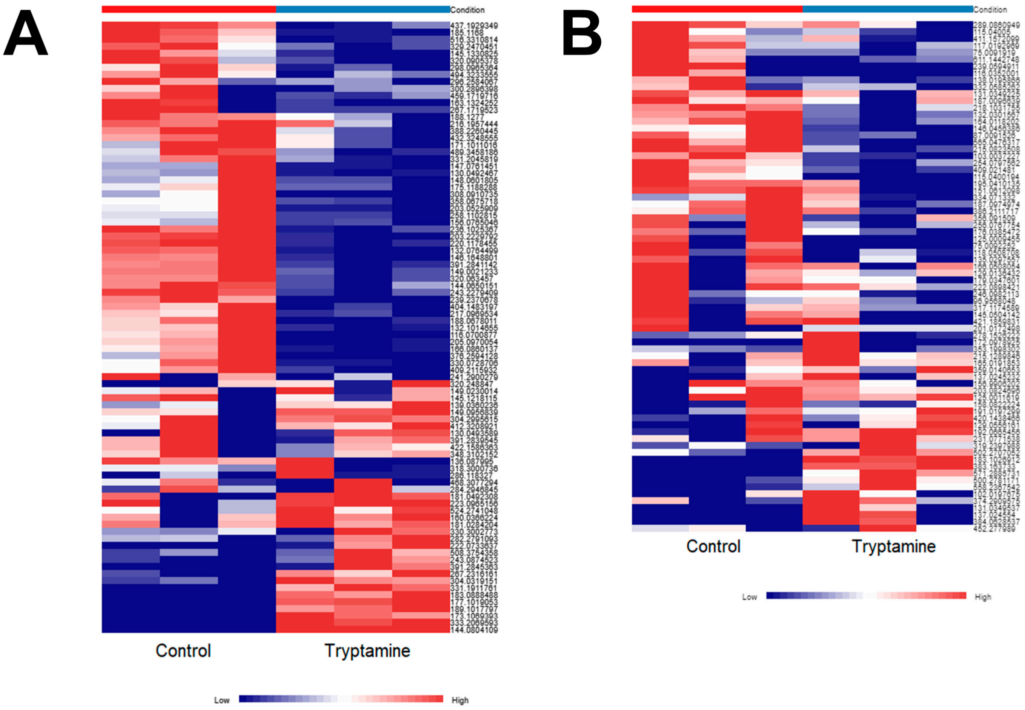

Figure S3. Heatmap overview of altered PC-3 cell metabolic profiles in LC-MS (A) positive and (B) negative ion modes before and after tryptamine treatment.

Arginine

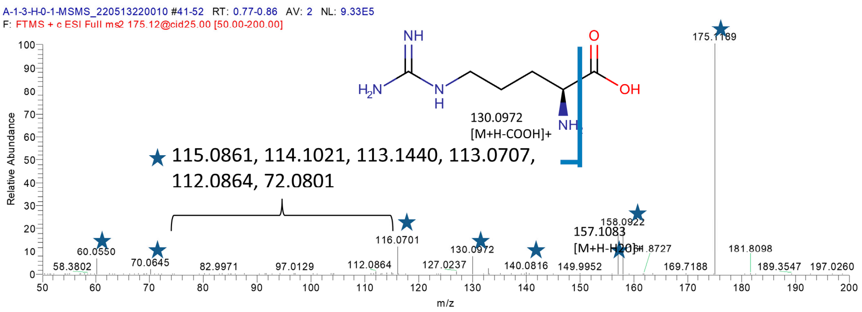

Proline

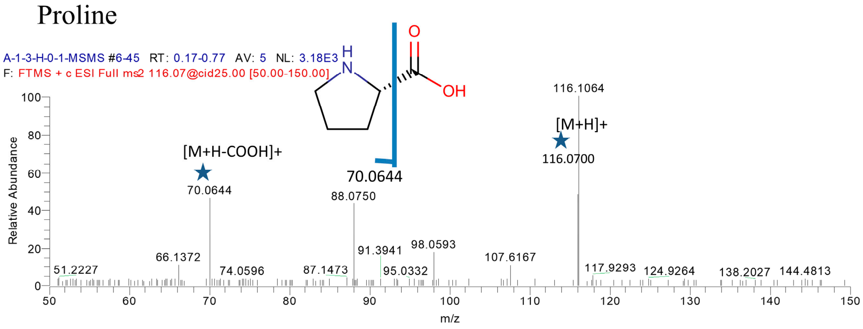

Creatine

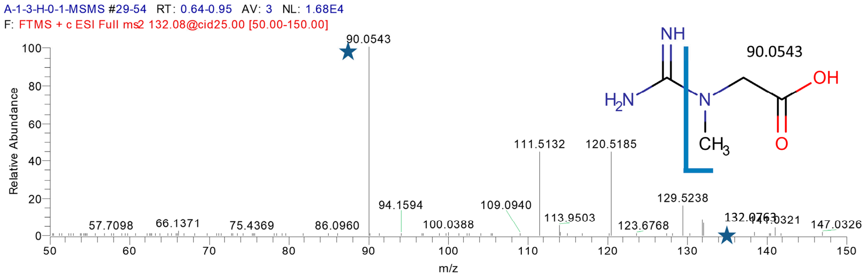

Spermidine

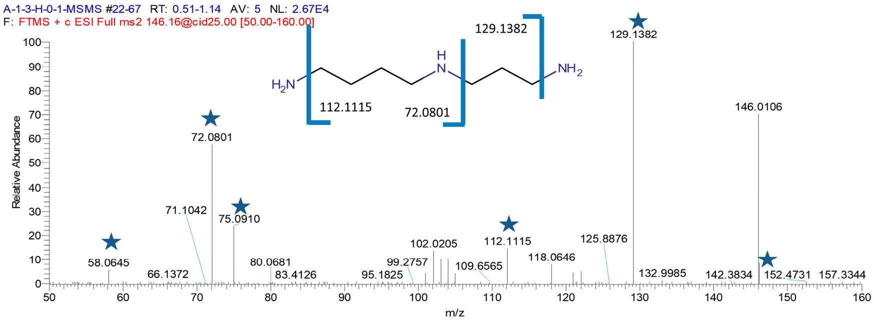

Spermine

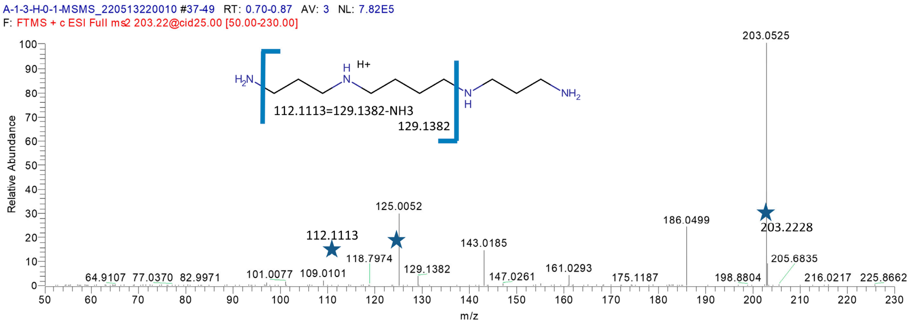

Serotonin

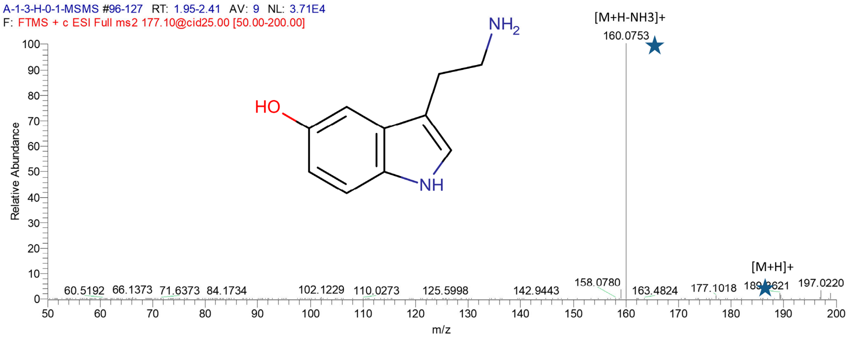

Aspartic acid

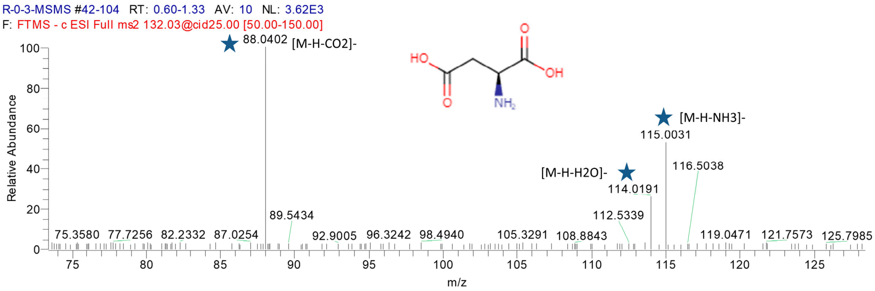

α-ketoisovaleric acid

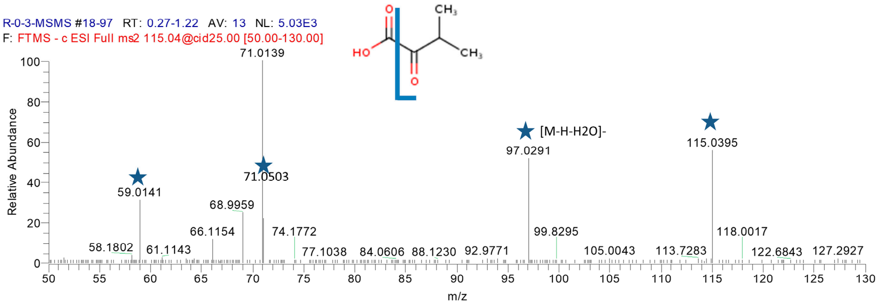

Pantothenic acid

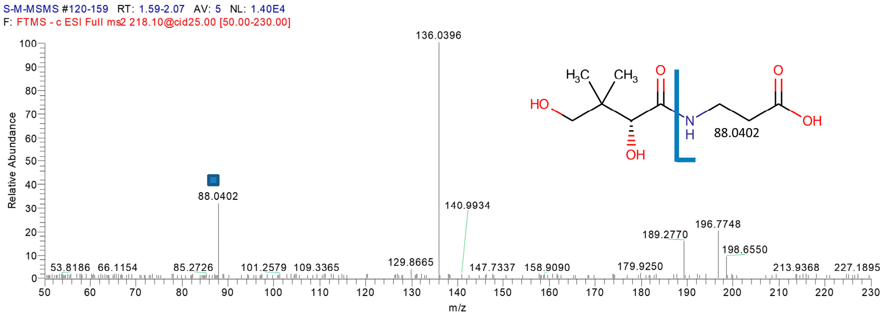

Ketoleucine

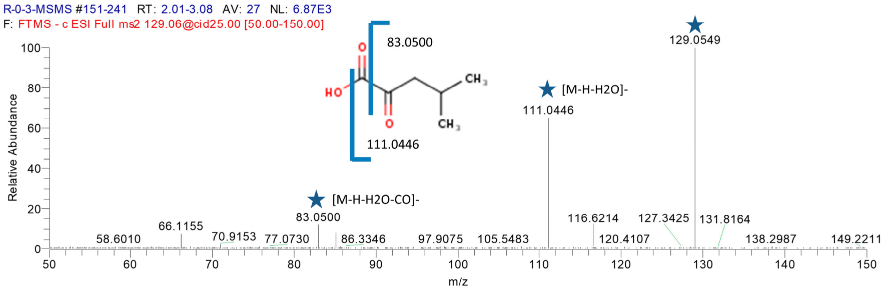

Figure S4 MS/MS analyses of the discriminant metabolites in panels.
